# Peripheral Blood Smear Demonstration of Lymphocyte Changes in Severe COVID-19

**DOI:** 10.4269/ajtmh.20-0721

**Published:** 2020-08-11

**Authors:** Chun-Tsu Lee, Winnie Z. Y. Teo

**Affiliations:** 1 Chronic and Fast Program, Alexandra Hospital, National University Hospital System, Singapore, Singapore;; 2 Department of Haematology-Oncology, National University Cancer Institute, Singapore (NCIS), National University Health System, Singapore, Singapore;; 3 Department of Laboratory Medicine, Alexandra Hospital, National University Hospital System, Singapore, Singapore;; 4 Department of Medicine, Yong Loo Lin School of Medicine, National University of Singapore, Singapore, Singapore

**Figure 1.**
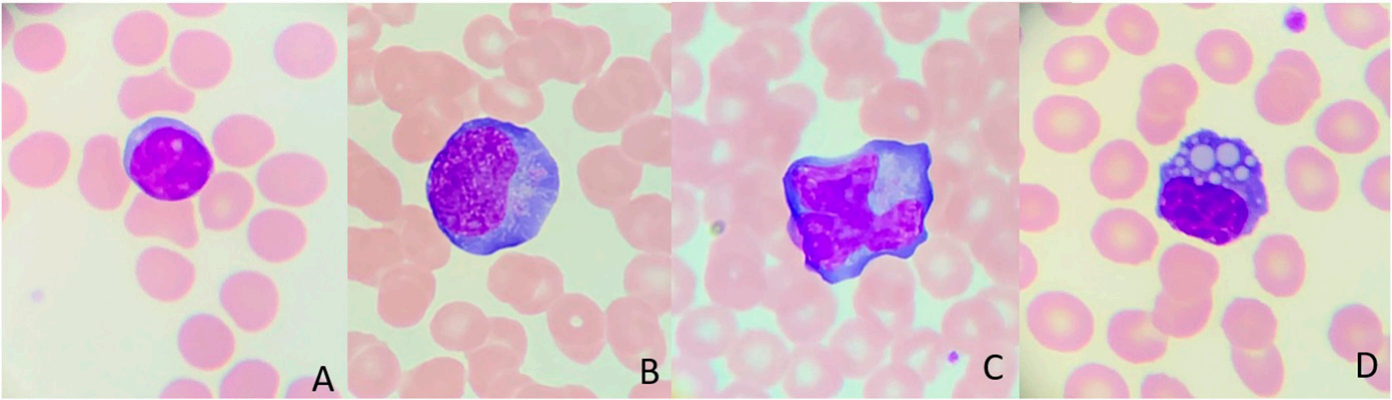
(**A**) Peripheral blood smear revealed a normal resting lymphocyte from healthy individual (Wright–Giemsa staining; viewed under oil immersion lens at ×1,000 magnification). (**B** and **C**) Peripheral blood smears from a patient with severe COVID-19 revealed an enlarged reactive lymphocyte with dark basophilic cytoplasms with peripheral accentuation, eccentric round or indented nucleus with dense chromatin, and perinuclear Hof. They have copious cytoplasms that scallop around adjacent red blood cells. They are generally seen in viral infections such as dengue fever, and infectious mononucleosis. (**D**) This is a Mott cell, a variant of plasma cell with immunoglobulin entrapped in the endoplasmic reticulum, in a form of Russell bodies (Wright–Giemsa staining; viewed under oil immersion lens at ×1,000 magnification).

A 60-year-old man with type two diabetes mellitus and ischemic heart disease came to the emergency department complaining of a 4-day history of unrelenting fever and cough. He was hemodynamically stable with a normal respiratory examination. Chest radiograph showed ground-glass opacities in the lower zones, and COVID-19 RNA PCR nasopharyngeal swab was positive. His full blood count showed a leukocyte count of 2.4 × 10^9^/L, hemoglobin of 16.2 g/L, platelet of 152 × 10^9^/L, and a low absolute lymphocyte of 0.8 × 10^9^/L. In addition, ferritin and C-reactive protein (CRP) were elevated at 850 ng/mL and 258 mg/L, respectively. Blood smears showed lymphoplasmacytoid lymphocytes with a round eccentric nucleus, deeply basophilic cytoplasm, and a perinuclear clear area (perinuclear hof) denoting the Golgi apparatus (Figure 1B). Reactive lymphocytes (Figure 1C) with abundant cytoplasm scalloping around the neighboring cells imparting a ballerina skirt appearance were encountered. Plasma cells with intracellular inclusions of immunoglobulins (Russell bodies), known as Mott cells, were seen in the blood film. Two days later, his condition deteriorated with severe hypoxemic respiratory failure requiring mechanical ventilation. Repeat chest radiograph showed diffuse pulmonary infiltrations in keeping with acute respiratory distress syndrome (ARDS).

During the incubation period of COVID-19, adaptive immunity plays a crucial role in eliminating the virus. In immunocompromised patients, because of medical comorbidities, an effective antiviral immunity cannot be mounted, leading to a hyperinflammatory state that culminates into ARDS.^1^ This case illustrates the morphological evolution of lymphocyte activation seen in a patient with COVID-19. During viral infection, B-lymphocytes are activated to become lymphoplasmacytoid lymphocytes and immunoglobulin-secreting plasma cells which have a distinctive morphology.^2–4^ There is emerging evidence that, in addition to elevated inflammatory markers and lymphopenia, elevated lymphoplasmacytoid lymphocytes, which correlate with antibody secreting and CD38^+^ antigen secreting B-lymphocytes, may predict clinical severity in COVID-19.^4,5^
